# Growing of the Cretan Therapeutic Herb *Origanum Dictamnus* in The Urban Fabric: The Effect of Substrate and Cultivation Site on Plant Growth and Potential Toxic Element Accumulation

**DOI:** 10.3390/plants12020336

**Published:** 2023-01-11

**Authors:** Aikaterini N. Martini, Maria Papafotiou, Ioannis Massas, Nikoleta Chorianopoulou

**Affiliations:** 1 Laboratory of Floriculture and Landscape Architecture, Department of Crop Science, School of Plant Science, Agricultural University of Athens, Iera Odos 75, 118 55 Athens, Greece; 2 Laboratory of Soil Science and Agricultural Chemistry, Department of Natural Resources and Agricultural Engineering, School of Plant Science, Agricultural University of Athens, Iera Odos 75, 118 55 Athens, Greece

**Keywords:** Cretan dittany, food safety, green roof, heavy metals, leaf washing, native ornamental plant, nutrients, roadside cultivation, soil vs. soilless substrate, urban agriculture

## Abstract

*Origanum dictamnus* L. (Lamiaceae) is a perennial herb endemic to the Greek island of Crete, widely used for tea preparation, medicinal purposes, and food flavoring, as well as an ornamental plant. The aim of this work was to introduce the species to the green roof sector while serving urban agriculture. Thus, its growth potential was investigated, along with the content of nutrients (N, P, K, Na) and the accumulation of heavy metals (Cu, Pb, Ni, Mn, Zn, Fe) in its tissues, in two cultivation sites in Athens, Greece, i.e., an extensive green roof and at ground level next to a moderate traffic road. Cultivation took place in plastic containers with a green roof infrastructure fitted, in two substrate types (grape marc compost, perlite, and pumice 3:3:4 *v*/*v*, and grape marc compost, perlite, pumice, and soil 3:3:2:2 *v*/*v*), with 10 cm depth. Plant growth was favored by the soil substrate, but it was also satisfactory in the soilless one. Cultivation site affected heavy metal accumulation, resulting in higher concentrations both in leaves and in flowers at street level, while no differences were observed in roots. Washing the tissues reduced heavy metal concentrations only in leaves produced at the street level. Substrate type significantly affected Mn concentration in all plant tissues and Fe in roots, with the highest values measured in the soil substrate. Thus, *O. dictamnus* could be effectively cultivated in sustainable green roofs, better on a soilless substrate to lower construction weight. Careful selection of the cultivation site could minimize contamination with environmental pollutants if human consumption is also desired.

## 1. Introduction

*Origanum dictamnus* L. (Lamiaceae), Cretan dittany, is a rare perennial plant, endemic to the Greek island of Crete. It is a densely white-woolly subshrub that grows 20–30 cm high, with round leaves of velvety texture bearing lilac flowers surrounded by purple-pink bracts in summer. It grows on mountainous rocky cliffs and gorges generally shaded or semi-shaded [1,2]. The plant is referred in the Greek mythology and is known since antiquity for its therapeutic properties mentioned by the Greek physician Hippocrates, the philosopher Aristotle, and the scholar and philosopher Theophrastus. The main constituents of its essential oil are carvacrol, γ-terpinene, p-cymene, linalool, and caryophyllene [3], and possess antimicrobial, antioxidant, and anti-ulcer pharmacological properties [3,4,5], as well as antiviral activity against influenza, viruses, and rhinoviruses [6]. *O. dictamnus* is classified as vulnerable on the IUCN Red List of Threatened Plant Species 1997 [7] and is protected by European legislation so that it does not become extinct.

*O. dictamnus* is cultivated in Crete and used in natural beauty products, as a culinary plant [8], and as a traditional medicinal plant, mainly as a tea plant, since its aerial parts have healing, astringent, and soothing properties [4,8]. In addition, *O. dictamnus* is used as an ornamental plant suitable for xeriscaping and has been proposed for use in urban extensive green roofs in semi-arid regions with limited irrigation supporting sustainability of the urban environment [9,10,11].

The population of cities is constantly increasing and the forecast for the coming years is that this will continue [12]. Cities are expanding at the expense of peri-urban agriculture, which is steadily declining as modern rapid transportation and the development of logistics make it easier to transport food from remote agricultural areas to urban centers. However, this strategy raises issues of food safety and, above all, of increasing the carbon footprint. Furthermore, the complex process of sourcing and distributing food from afar often proves vulnerable to crises, such as the COVID-19 pandemic [13]. The development of urban agriculture leads to shorter supply chains and a reduction in logistics networks contributing to a reduction in the use of fossil fuels, potentially reducing food waste due to reduced losses and therefore supporting low carbon economic strategies [14,15,16].

The lack of urban land for soil cultivation, or the high monetary value of urban land, as well as the contamination of the soil by urban pollutants, leads to the exploitation of alternative sites for the implementation of urban agriculture, such as abandoned buildings for the implementation of totally controlled cultivation systems (urban farms involving hydroponics and led lighting) or building roofs.

In the case of the utilization of roofs, the benefits multiply, with the multiple services of green roofs to the sustainability of the urban environment, such as storm water management, reduction of urban heat island effects, noise reduction, and building temperature regulation, as well as connection between the built and the natural environment and direct contact of building occupants with the green roof with social and educational benefits [17,18,19,20].

Cultivation in urban green roofs of perennial plants with dual character, i.e., with ornamental and commercial value, such as *O. dictamnus*, is an interesting combination of green roof technology for environmental benefits with urban agriculture [21,22]. Furthermore, in semi-arid regions with limited water availability for urban green use and in view of the climate crisis, which is expected to exacerbate drought and heat conditions on green roofs, the need to explore the possibility of using native species including herbs with limited water needs and with tradition of use by the local community, is particularly attractive in the context of sustainability of the city [21,22,23,24].

Urban agriculture, however, also involves the risk of contamination of cultivated plants with pollutants produced by human activity in the urban environment. Soil, air, and water pollution with toxic elements poses a nutritional risk [25,26,27,28,29]. The use of fossil fuels for heating and vehicle movement in cities is still very high, burdening the urban environment with pollutants, which are deposited on the plant surface or soil and then absorbed by plant tissues [30,31]. The degree of risk from the consumption of urban agriculture products depends on several factors including the location and method of cultivation, the plant species, the part of the plant consumed, and the type of potentially toxic element [21,32,33,34]. Usually, inflorescences and fruits accumulate lower amounts of heavy metals than leaves and underground organs [33,34,35]. Consuming raw plant tissue of medicinal plants grown in contaminated environments can be dangerous [36], while the method of processing has been shown to be important in reducing the risk, as, for example, boiling the medicinal plant in water results in the extraction of higher levels of the heavy metals than immersion in hot water [37].

Proximity of crops to pollution sources increased the accumulation of heavy metals in plant tissues, as did cultivation in soil compared to soilless planting systems [21,38]. Roadside cultivation can be significantly influenced by road proximity due to road dust particles [39,40,41] and higher overall traffic increases the trace element content in crop biomass, while the presence of barriers between the road and the growing site can significantly reduce the heavy metal content in tissues [42]. The variability in heavy metal content in different plant species and plant tissues, as well as in cultivation sites and systems, highlights the importance of specialized monitoring per crop and place, to assess the potential risk to human health from cultivation in the urban environment.

Thus, the aim of this work was to evaluate the growth and safety of *O. dictamnus* for human consumption, when cultivated in different substrates and sites in the urban fabric. Plants were cultivated on a building roof or next to a moderate traffic road on a soilless substrate or on a substrate amended with soil, and data were recorded as for their growth and flowering, nutrient content, and heavy metal accumulation in aboveground plant parts and in the roots. Canopy washing was also examined as a way to remove surface deposition of environmental pollutants and thereby its effect on heavy metal concentration.

## 2. Results

### 2.1. Plant Growth

**Figure 1:**
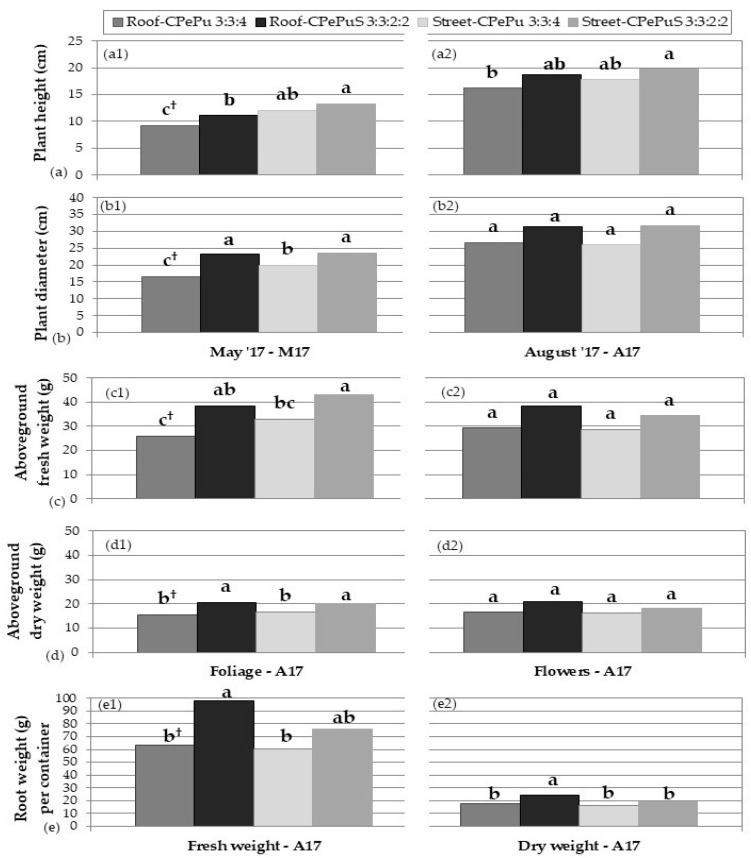
Effect of cultivation site and substrate type on growth of *O. dictamnus*, after growing for five and eight months in plastic containers with a green roof infrastructure fitted. Μ17 = late May 2017; A17 = late August 2017. (**a1**,**a2**): plant height in May and August, respectively; (**b1**,**b2**): plant diameter in May and August, respectively; (**c1**): foliage fresh weight; (**c2**): flower fresh weight; (**d1**): foliage dry weight; (**d2**): flower dry weight; (**e1**,**e2**): root fresh and dry weight, respectively. ^†^ Mean values (*n* = 12, excepting roots in which *n* = 6) in each bar followed by the same lower-case letter do not differ significantly at *p* ≤ 0.05 by Student’s *t* test. C: grape marc compost, Pe: perlite, Pu: pumice, S: soil.

**Figure 2:**
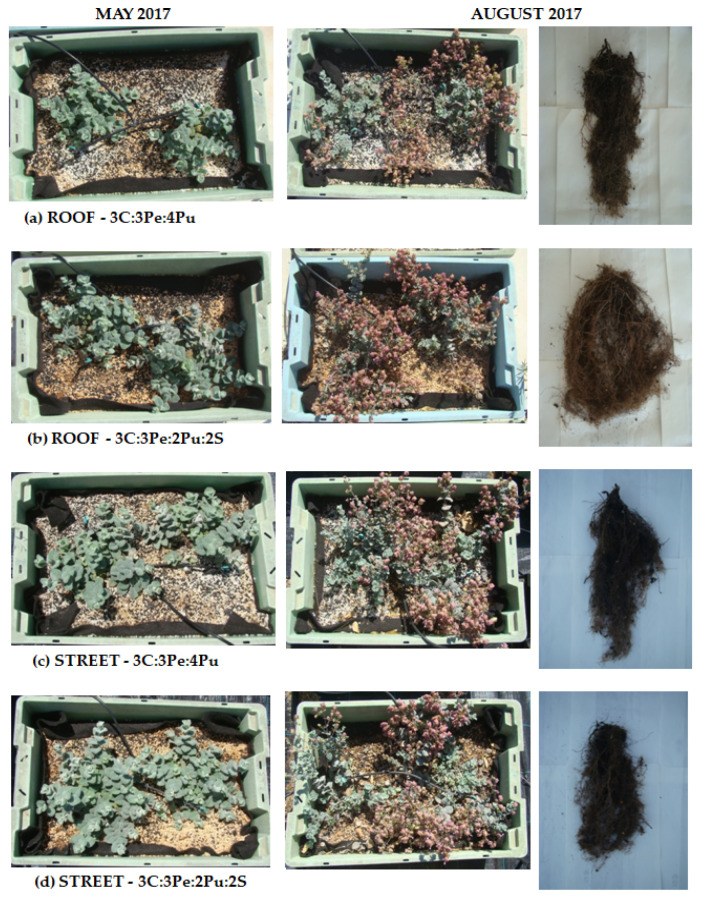
Characteristic aboveground and root system growth of *Origanum dictamnus* cultivated in marked substrate type and cultivation site, for five (May 2017) and eight months (August 2017).

Five months after planting, at the end of May 2017, before flowering, two-way ANOVA showed that plant height and diameter were affected by both experimental factors (Table 1). Plant height was promoted by soil substrate and cultivation at the street level (Figure 1(a1)). In addition, plants grown in the soil substrate had a larger diameter than those in the soilless one, regardless of cultivation site (Figure 1(b1) and Figure 2). Especially plants grown in the soilless substrate in the roof had an even smaller diameter (Figure 1(b1) and Figure 2a).

Eight months after planting, at the end of August 2017, when flowering of the plants was almost complete, the substrate type ceased to have a significant effect on aboveground plant growth, while cultivation site affected both plant height and diameter and plants were still taller at the street level than on the roof (Table 1, Figure 1(a2,b2) and Figure 2). Flowering started in June and continued until August, with no differences in the number of flowering shoots between treatments throughout the flowering period [43]. Foliage fresh and dry weight were only affected by substrate type, both being greater in soil substrate (Table 1, Figure 1(c1,d1)). Regarding the fresh and dry weight of flowers, no statistically significant differences were found between experimental factors or treatments (Table 1, Figure 1(c2,d2)). Root fresh weight per container was only affected by substrate type, being greater in the soil substrate especially in the roof (Table 1, Figure 1(e1)), while root dry weight per container was affected both by substrate and cultivation site and was greatest in plants grown on the roof, in soil substrate (Table 1, Figure 1(e2) and Figure 2).

### 2.2. Nutrient Content

Regarding three-way ANOVA of nutrient concentrations in leaves and flowers, no interactions were found between washing and the other two factors, i.e., cultivation site and substrate type (three-way ANOVA results not presented). Furthermore, washing had no significant effect on any nutrient concentration. Thus, concentrations of nutrients were analyzed together for washed and unwashed leaves and flowers using two-way ANOVA, with the main factors being cultivation site and substrate type.

Percentage of N did not differ between treatments in leaves and flowers, whereas roots at the street level had higher N than those produced in the roof (Table 2). Flowers contained less N than the other tissues (Table 2B). Percentage of P in leaves was highest in the roof in the soilless substrate, while there were no differences between treatments in flowers and roots (Table 2). In all tissues, levels of K were higher in the soilless substrate than in the soil substrate (Table 2). Roots contained less P and K than the other tissues (Table 2C). The highest values of Na were found at the street level in the soilless substrate for all tissues (Table 2). Roots contained the most Na of all tissues (Table 2C).

### 2.3. Heavy Metal Accumulation

Chemical analysis of substrate components showed that all studied micronutrients (Cu, Mn, Fe, Zn), extracted by DTPA, were in deficiency, while concentrations of Pb and Ni were low and indicative of very low concentrations available for plant uptake (Table 3).

Three-way ANOVA of heavy metal concentrations both in leaves and flowers showed significant interactions between cultivation site and washing in most heavy metals, except in Cu and Ni (Table 4). Although washing decreased the concentration of Cu and Ni in leaves, it had no significant effect on the concentration of Cu and Ni in flowers (Table 4). Thus, heavy metal concentrations were analyzed separately for washed and unwashed leaves and flowers using two-way ANOVA, with cultivation site and substrate type as the main factors. Corresponding two-way ANOVA was applied to the roots, since all root samples were washed to remove substrate from the root system.

After eight months of cultivation, the concentration of Cu was higher both in leaves and flowers produced at the street level compared to those on the roof, regardless of substrate type (Table 4). Washing also affected Cu concentration in leaves, reducing its concentration (Table 4 and Table 5). Concentrations of Pb and Ni were higher in leaves produced at street level compared to those on the roof, while in flowers there were no differences between treatments (Table 4 and Table 5). Concentrations of Mn were the only ones affected by substrate type, being higher in leaves and flowers produced in the soil substrate than in the soilless one (Table 5). In unwashed leaves and flowers, cultivation site also affected Mn concentrations, which were higher at the street level compared to the roof (Table 5). Concentration of Zn was higher in leaves and flowers produced at the street level compared to those on the roof, while washing reduced its concentration in leaves and flowers produced at street level (Table 4 and Table 5). Concentration of Fe was higher in leaves and flowers produced at street level compared to those on the roof, while washing reduced Fe concentration (Table 4 and Table 5). Flowers from plants grown at street level contained lower concentrations of heavy metals compared to the corresponding leaves, whereas leaves and flowers grown on the roof had equal heavy metal concentrations (Table 5).

In roots, concentrations of Cu, Pb, Ni, and Zn did not differ between treatments, while soil substrate resulted in higher Mn and Fe concentrations compared to soilless one (Table 6).

Comparing roots, leaves, and flowers in regards to heavy metal accumulation, Cu, Ni and Mn concentrations were higher in roots than in leaves and flowers, except for Cu concentrations in the roots produced at street level that were similar to those in leaves and flowers, while there were no differences in Pb and Zn concentrations between the three different types of tissues (Table 5 and Table 6). Extremely higher concentrations of Fe were found in roots than in leaves and flowers, about 2–3 times higher in the soilless substrate and 4–7 times higher in the soil substrate (Table 5 and Table 6).

## 3. Discussion

### 3.1. Plant Growth

The effect of experimental factors, i.e., cultivation site and substrate type, on plant growth depended on the plant developmental stage and/or plant age. Before flowering, five months after planting, plant diameter and height were promoted by soil substrate and cultivation at the street level, while after flowering, only the cultivation site still had an effect resulting in taller plants. Fresh and dry weights of foliage and root systems at the end of the experiment, after the eight-month growth period, were favored by the soil substrate, whereas flower weights did not differ among treatments, while root dry weight was greatest in plants grown in the soil substrate on the roof. The diameter of *O. dictamnus* has been shown to benefit by a soil substrate during its initial growth at the establishment stage as well [10,45], while *C. maritimum* in a similar experiment had the same response as *O. dictamnus* in the present study [21]. Similarly, other Mediterranean xerophytes, such as *Convonvulus cneorum* and *Sideritis athoa*, showed faster growth when in a substrate containing soil in the first months of their cultivation on an urban extensive green roof [45]. The reduced plant height and the increased root dry weight of *O. dictamnus* plants grown in the roof could be attributed to adverse conditions of the roof, as a mechanism to avoid the dry-hot and windy roof conditions [46,47,48]. Nevertheless, plants grew satisfactorily on the roof as well, with no differences in flowering compared to plants grown at the street level, as has already been reported [43].

Taking into consideration that although foliar and root growth was greater in the soil substrate, the general plant growth was also satisfactory, and flowering remained unaffected in the soilless one; therefore, the soilless substrate could be recommended for green roof cultivation in order to achieve reduced construction weight. This is in accordance with other studies on *O. dictamnus* [10,43,49] or other Mediterranean xerophytes [21,22,50,51], in which lightweight and highly porous soilless substrates were recommended for green roof cultivation.

### 3.2. Nutrient Content

Νo previous work was found about the nutrient content of *O. dictamnus* tissues. In this work, the percentage of N was affected neither by cultivation site nor by substrate type, except in roots in which the percentage of N was higher at the street level. Neither the percentage of P was affected by the experimental factors or treatments, except in the percentage of P in leaves, which was highest in the roof in the soilless substrate, confirmed in a similar work on *C. maritimum* where the concentration of P was favored by the cultivation on the green roof [21]. Additionally, levels of K were affected by substrate type, being higher in the soilless substrate for all plant tissues. As for Na, its values were affected by both experimental factors, being higher at the street level in the soilless substrate for all plant tissues. Similarly, *C. maritimum* cultivation at the street level induced higher Na concentrations in tissues compared to the green roof [21].

### 3.3. Heavy Metal Accumulation

Chemical analysis of substrate components showed that all studied heavy metals were low and indicative of very low concentrations available for plant uptake.

Three-way ANOVA of heavy metal concentrations both in leaves and flowers showed significant interactions between cultivation site and washing in most heavy metals, except in Cu and Ni. Washing decreased the concentration of Cu and Ni in leaves but had no significant effect on their concentrations in flowers.

Cultivation site significantly affected concentrations of Cu, Pb, Ni, Zn, and Fe in leaves and Cu, Zn, and Fe in flowers, which were all higher at the street level compared to the roof. On the contrary, concentrations of all heavy metals in roots were not affected by cultivation site, except Cu, whose concentration was higher at the street level too. Simultaneously with the present experiment, a similar experiment with *C. maritimum* was carried out side by side. In *C. maritimum,* which has a thick rhizomatous root system, instead of the fine tufted root system of *O. dictamnus*, the cultivation site affected the accumulation of Pb and Zn, with Pb concentration being higher by the street, while Zn was higher on the roof [21].

The effect of substrate type on heavy metal accumulation depended on plant tissue. In leaves and flowers, substrate type affected only Mn concentration, while in roots, it affected Mn and Fe concentrations. In all tissues, higher values of Mn or Fe (in the case of roots) were recorded in the soil substrate. Similar results were found in *C. maritimum* leaves regarding Mn concentration, but in its roots, substrate type affected Ni, Zn, and Fe accumulation, with Ni and Zn being higher in the soilless substrate and only Fe being higher in the soil one [21], as found for Fe in the present study.

Despite the interactions between washing and cultivation site in their effect on heavy metal accumulation, it was mentioned above that washing reduced significantly the concentration of Cu and Ni in leaves. Comparing washed and unwashed leaves, it can be seen that leaves at the street level had lower concentrations of heavy metals when washed, but this was not enough in order to reduce their concentration under the maximum acceptable limits according to FAO/WHO [44]. In corresponding flowers, produced at street level, only a slight reduction in heavy metal accumulation was succeeded by washing. As regards Fe, washing reduced its concentration in both plant tissues. The effectiveness of washing in reducing heavy metal accumulation in *O. dictamnus* contrasts with what was found in *C. maritimum*, where washing did not reduce heavy metal concentrations [21], probably because its smooth fleshy leaves were holding less dust and settled environmental atmospheric pollutants compared to the extremely hairy leaves of *O. dictamnus*. Nevertheless, in both species, washing of tissues proved ineffective in the reduction of heavy metals concentration, under the permissible limits [44]. This indicates that the measured concentrations of heavy metals were not due to the dust held by the foliage, but that the deposited environmental pollutants had been absorbed by the plant tissues. There are bibliographic reports related to the effect of washing on concentrations of heavy metals, mainly on leafy vegetables, that showed no differences between unwashed and washed leaves in the urban area [52,53], while others revealed a higher concentration of toxic elements in unwashed samples than the washed samples [27,54]. Thus, the effectiveness of washing in reducing heavy metal concentration in edible plant parts could vary according to the metal [31] and the plant species.

Comparing the three types of *O. dictamnus* plant tissues regarding the accumulation of heavy metals, flowers contained fewer heavy metals than leaves when they were produced at street level, but no differences were present between these two tissues when they were produced in the roof. This variation of leaves and flowers with respect to the site of cultivation could be attributed to higher concentrations of pollutants at street level that allowed differences to appear, to the presence of dense hairs on the leaves that facilitate dust settling and absorption of environmental pollutants, and the fact that the leaves formed earlier than the flowers, which were present on the plant only during the period June—August. Trichomes and cuticular waxes were shown to retain airborne particulate matter deposited on plant leaves [55], followed by heavy metals direct absorption through stomata [31]. The way initial uptake by the leaf will be subsequently translocated throughout the plant is dependent on various factors, such as leaf anatomy (i.e., stomatal index, trichome density and length, and leaf maturity) and chemical characteristics of the metal adsorbed [56,57]. When roots were included in the comparison, concentrations of Cu (only in tissues produced in the roof), Ni, Mn, and particularly of Fe in roots were higher than those recorded in the leaves or flowers. In *C. maritimum*, the Fe concentration in roots was also significantly higher than that recorded in leaves [21]. It is very difficult to distinguish whether the concentration of metals in plant tissues is taken up by root cells from the substrate or by leaf surfaces from the atmosphere, because the two kinds of uptake pathways can occur simultaneously near urban and industrial areas, while the major portion of absorbed metals (more than 95%) is stored in the plant tissue that did the uptake [58].

Comparing recorded concentrations of heavy metals with maximum acceptable limits according to FAO/WHO [44], it was found that concentration of Pb, Ni, and Mn in all plant tissues, as well as those of Zn in leaves and roots, surpassed the limits, making unsafe for human consumption tissues of *O. dictamnus* produced in the region of the Agricultural University of Athens. Both cultivation sites were next to Iera Odos street, which is a moderate traffic road in the city of Athens, without any building or vegetation between the street and the cultivation sites, which could serve as a barrier to traffic-related pollutants. The high concentrations of heavy metals in both sites could be attributed to both the polluted urban atmosphere and the particular road traffic including trucks, as there are several warehouses in the area, in verification of Säumel et al. [42], who showed that overall higher traffic increases heavy metal content in crop biomass within the urban fabric. Antisari et al. [38] also concluded that the concentration of heavy metals in urban-grown vegetables is strictly related to the site in the city where plants are grown, resulting in increased risks of heavy metal accumulation, when plants are cultivated nearby pollution sources. Replacing more than 50% of old technology vehicles with new technology vehicles and especially electric vehicles is a promising solution for reducing air pollution in cities [59,60], thus making urban agriculture safer for humans. At the Agricultural University of Athens, concentrations of Pb, Ni, and Mn in leaf tissues of *C. maritimum* [21], as well as concentrations of Pb and Ni in leaves of *Salvia officinalis* [40] and *Origanum vulgare* ssp. *hirtum* [41] were higher than the permitted levels in agreement with our results on *O. dictamnus*, independent of fertilization, substrate type, and whether cultivation was next to Iera Odos street or on the nearby second floor roof.

Although a high accumulation of Pb due to atmospheric deposition was also found in leafy vegetables [27] and wild edible mushrooms [61], collected from the urban environment, there are some reports showing that crop plants cultivated in urban gardens, including rooftop gardens, contained heavy metals within the permissible limits [27,53,54]. Moreover, aromatic and medicinal plants can be grown as alternative high-value crops in metal-polluted areas in order to produce essential oils, which would be a metal-free marketable final product [62,63].

## 4. Materials and Methods

### 4.1. Experimental Set-Up (Plant Material, Cultivation System, and Site)

Six-week-old *O. dictamnus* plants produced by cuttings were planted in late December 2016 in 40 cm × 60 cm × 22 cm plastic containers (two plants per container planted diagonally) with 10 cm deep substrate. The containers had a green roof infrastructure fitted (moisture retention and protection of the insulation mat FLW-500, drainage layer Diadrain-25H, and filter sheet VLF-150; Landco Ltd., green roof systems Diadem, Athens, Greece).

The containers were placed in two cultivation sites on the campus of the Agricultural University of Athens, half of them on a fully exposed second-floor flat roof of a building adjacent to Iera Odos street (37°59′01 N, 23°42′19 E, approximate height 7 m, approximate distance of the building from the street 12 m), and the other half at ground level in an open field next to Iera Odos street (37°59′03 N, 23°42′08 E, pavement width 1.5 m). Iera Odos street is a moderate traffic road in the city of Athens, Greece. No buildings or vegetation served as barriers to traffic-related pollutants between the street and the cultivation sites.

The containers were placed following the completely randomized design. A factorial experiment was carried out with the cultivation site (green roof, street level) and substrate type (with soil, soilless) as factors. Therefore, four treatments were applied (two cultivation sites × two substrate types), and in each treatment six containers were used, with two plants per container (*n* values are shown in data tables and figures).

### 4.2. Substrate

Two types of substrates were used; one consisting of grape marc compost (C), perlite (Pe), and pumice (Pu) (3:3:4, *v*/*v*, soilless substrate) and one of grape marc compost, perlite, pumice, and soil (S) (3:3:2:2, *v*/*v*, soil substrate). The grape marc compost was produced in the field of the Agricultural University of Athens by a process, which is routinely used for composting grape marc in Greece [21,64]. The grape marc compost had a pH of 6.45 and EC 1155 µS/cm, the perlite particles were 1–5 mm in diameter (Perloflor; ISOCON S.A., Athens, Greece); the pumice particles were 1–8 mm in diameter (LAVA Mining and Quarrying Co., Paiania, Attiki, Greece) and the soil contained 21.4% clay, 25.8% silt, 52.8% sand, and 21.32% equivalent CaCO_3_, and had a pH of 7.9 and EC 241 µS/cm. The chemical composition of the substrate components is shown in Table 2. The two substrates had similar pH values (7.5–7.6), while the EC value was 267 µS/cm for the soil substrate and 352 µS/cm for the soilless type. Detailed physicochemical properties of the substrates are given in [65].

### 4.3. Irrigation

Irrigation was applied from April to August. Automatic drip irrigation on the substrate surface was applied before sunrise by two drippers placed at equal distances from the center of the container and the plants. The dripper supply was 4 L·h^–1^ and the duration of each irrigation event was 35 min, sufficient to allow water to drain off the container.

Plants were irrigated when the substrate moisture was 17–20% *v*/*v*. In the first week of each month, substrate moisture (% *v*/*v*) was recorded daily to check the need to adjust the irrigation schedule. Three measurements from each container at 19:00 to 20:00 HR were taken using a handheld moisture meter (HH2; Delta-T devices, Cambridge, UK), with a soil moisture dielectric sensor (WET-2; Delta-T devices) inserted from the substrate surface, which measured 65 mm in depth and 45 mm in width. Therefore, irrigation was scheduled every four days from April to mid-July and every three days mid-July to August.

### 4.4. Meteorological Data

**Figure 3:**
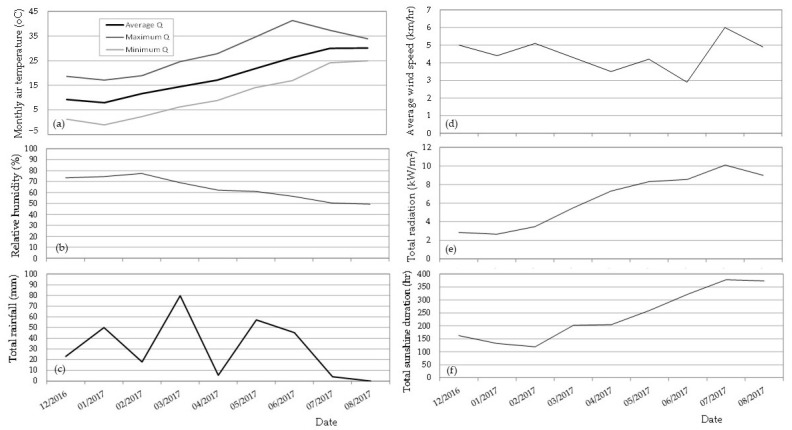
Average, maximum, and minimum monthly air temperature (**a**), average monthly relative humidity (**b**), total monthly rainfall (**c**), average monthly wind speed (**d**), total monthly radiation (**e**), and total monthly sunshine duration (**f**), during the experimental period (December 2016 to August 2017).

The monthly average, maximum and minimum air temperature, the total rainfall, and the average wind speed (http://meteosearch.meteo.gr/, accessed on 7 March 2022), the monthly average relative humidity and total radiation (Laboratory of General and Agricultural Meteorology, Agricultural University of Athens), and the monthly total sunshine duration (http://www.emy.gr/emy/el/climatology/climatology, accessed on 30 March 2022) during the experimental period (December 2016 to August 2017) are presented in Figure 3.

### 4.5. Plant Growth Evaluation

Plant growth was recorded monthly by measuring canopy height and horizontal diameter (average of larger horizontal diameter and its perpendicular). In the present study, growth data recorded in May and August are presented to show plant growth during the fifth and eighth month, before and after flowering, respectively. At the end of the experiment (end of August 2017), the fresh and dry weight of the aboveground part of each plant, separately for foliage and flowers, and of the root system were also recorded (see below in Section 4.6).

### 4.6. Heavy Metal and Nutrient Determination

At the beginning of September 2017, before the first rainfall, the aboveground part of the plants, foliage, and flowers separately, was collected to determine the accumulation of heavy metals and nutrient concentrations in the leaves and flowers, as in this period (end of summer) they are harvested for human consumption. The root system of the plants was also removed and rinsed under running tap water in a colander to reduce root loss. The roots of both plants of each container constituted one sample because the roots were tangled and difficult to separate. The fresh weights of the aboveground part and roots were measured immediately after collection. Then, half of the foliage and flower samples of each treatment were immersed in distilled water for 1 min and then rinsed under running tap water to wash off the dust deposited on the tissues. This was done to test whether washing could reduce potential heavy metal concentrations, in case heavy metals were found both on the leaf surface and inside the leaf tissue. The samples were then dried in an oven at 60 °C for 7 days and their dry weight was measured. In the dried foliage samples, the leaves were removed from the shoots and used for further analyses. Samples of washed and unwashed leaves and flowers and those of roots were crushed and ground in a mill (Retsch ZM1000, Apeldoorn, The Netherlands), followed by sieving through a 0.5 mm sieve. They were then placed in individual airtight plastic bags and kept refrigerated until analysis.

For the determination of heavy metals, a certain amount (1 g) of dried plant sample was placed on a porcelain crucible in a muffle furnace (at 550 °C for 3 h); to the combustion product, 5 mL HNO_3_ (65%) was added, the solution was filtered, and finally the filtrate was diluted with distilled water to a certain volume (25 mL). Concentrations of the heavy metals copper (Cu), lead (Pb), nickel (Ni), manganese (Mn), zinc (Zn), and iron (Fe) in samples were determined by atomic absorption spectrophotometry using a Varian-Spectra A300 system (Varian Inc., Palo Alto, CA, USA). To conclude whether the leaves and flowers were safe to consume, the recorded heavy metal concentrations were compared with the maximum acceptable limits for each heavy metal concentration in edible plants, i.e., 40.0 for Cu, 5.0 for Pb, 2.0 for Ni, 30.0 for Mn, and 60.0 for Zn (mg/kg dry matter), according to FAO/WHO [44]. The nitrogen (N) content of plant samples was determined by the Kjeldahl method in the Bucchi apparatus [66]. The phosphorus content of the plant samples was determined with a Shimadzu UV-1700 spectrophotometer (Shimadzu, Tokyo, Japan). For every 10 samples, a control sample was analyzed and at the end of the measurement process, 30% of the samples were reanalyzed to test reproducibility. Exchangeable potassium (K) and sodium (Na) concentrations were quantified using a PGI 2000 flame photometer (PG Instruments Ltd., Leicestershire, UK).

### 4.7. Statistical Analysis

The data followed the normal distribution. The significance of the experiment was tested by one-, two-, or three-way analysis of variance (ANOVA), and the treatment means were compared by Student’s *t* test at *p* ≤ 0.05 (JMP 13.0 software, SAS Institute Inc., Cary, NC, USA, 2013).

## 5. Conclusions

*O. dictamnus* grew well in the urban environment, both at the ground level next to a moderate traffic road and in an extensive green roof.

Plant canopy and root growth were favored by a soil substrate, although they were satisfactory in the soilless one as well. The soilless light weight substrate should be preferred in case of green roof cultivation, in order to lower construction weight.

Cultivation site affected heavy metal accumulation, resulting in higher concentrations of heavy metals both in leaves (for all elements) and flowers (except Pb and Ni) at street level compared to the roof, while no differences were observed in the roots.

Washing the tissues reduced heavy metal concentrations only in leaves produced at the street level.

Substrate type significantly affected Mn concentration in all plant tissues and Fe in roots, with the highest values measured in the soil substrate. Values of Fe in roots were multiple (4–18 times higher) of those recorded in leaves and flowers.

Concentrations of most heavy metals exceeded the permissible limits in all plant tissues at both cultivation sites, except Cu in all tissues and Zn in flowers, as well as leaves and roots, only when produced on the roof.

*O. dictamnus* could be effectively cultivated in sustainable green roofs, supporting urban horticulture, although the cultivation site should be carefully selected to minimize contamination with environmental pollutants, if human consumption of the edible plant parts is also desired.

## Figures and Tables

**Table 1:** Effect of experimental factors, i.e., cultivation site and substrate type, on growth of *O. dictamnus*, after growing for five and eight months in plastic containers with a green roof infrastructure fitted. Μ17 = late May 2017; A17 = late August 2017.

Significance ^§^ (Two-Way ANOVA)	Plant Height (cm) M17/ A17	Plant Diameter (cm) M17/ A17	Fresh Weight (g) Foliage/ Flowers A17	Dry Weight (g) Foliage/ Flowers A17	Root Weight (g) Per Container Fresh Weight/ Dry Weight A17
*F*_cultivation site_	**/ *	*/ **	NS/ NS	NS/ NS	NS/ *
*F*_substrate type_	**/ NS	**/ NS	**/ NS	**/ NS	*/ **
*F*_interaction_	NS/ NS	NS/ NS	NS/ NS	NS/ NS	NS/ NS
*F*_one-way ANOVA_	**/ *	**/ NS	**/ NS	**/ NS	*/ **

^§^ NS, *, or **, are non-significant at *p* ≤ 0.05, significant at *p* ≤ 0.05, or *p* ≤ 0.01, respectively.

**Table 2:** Effect of cultivation site and substrate type on content (%) of the nutrients nitrogen (N), phosphorus (P), potassium (K), and sodium (Na), in leaves (A), flowers (B), and roots (C) of *O. dictamnus*, collected after cultivation for eight months in plastic containers with a green roof infrastructure fitted. In leaves and flowers, data for washed and unwashed tissues are presented together.

**A. LEAVES**					
**Cultivation Site**	**Substrate Type (*v*/*v*)**	**N**	**P**	**K**	**Na**
Roof	3C:3Pe:4Pu	1.8 a ^†^	0.45 a	3.7 a	0.03 c
	3C:3Pe:2Pu:2S	1.7 a	0.32 b	2.5 b	0.03 c
Street	3C:3Pe:4Pu	1.8 a	0.36 b	3.3 a	0.12 a
	3C:3Pe:2Pu:2S	2.0 a	0.36 b	2.5 b	0.09 b
Significance ^§^					
*F*_cultivation site_		NS	-	NS	**
*F*_substrate type_		NS	-	**	NS
*F*_interaction_		NS	**	NS	NS
*F*_one-way ANOVA_		NS	**	**	**
**B. FLOWERS**					
**Cultivation Site**	**Substrate Type (*v*/*v*)**	**N**	**P**	**K**	**Na**
Roof	3C:3Pe:4Pu	1.2 a ^†^	0.34 a	3.3 a	0.02 b
	3C:3Pe:2Pu:2S	1.3 a	0.27 a	2.5 b	0.02 b
Street	3C:3Pe:4Pu	1.4 a	0.33 a	2.9 ab	0.11 a
	3C:3Pe:2Pu:2S	1.5 a	0.32 a	2.4 b	0.04 b
Significance ^§^					
*F*_cultivation site_		NS	NS	NS	-
*F*_substrate type_		NS	NS	**	-
*F*_interaction_		NS	NS	NS	**
*F*_one-way ANOVA_		NS	NS	**	**
**C. ROOTS**					
**Cultivation Site**	**Substrate Type (*v*/*v*)**	**N**	**P**	**K**	**Na**
Roof	3C:3Pe:4Pu	1.4 b ^†^	0.28 a	1.8 a	0.28 b
	3C:3Pe:2Pu:2S	1.5 b	0.20 a	0.8 c	0.12 c
Street	3C:3Pe:4Pu	1.8 a	0.23 a	1.2 b	0.47 a
	3C:3Pe:2Pu:2S	1.6 ab	0.20 a	0.7 c	0.30 b
Significance ^§^					
*F*_cultivation site_		*	NS	-	**
*F*_substrate type_		NS	NS	-	**
*F*_interaction_		NS	NS	**	NS
*F*_one-way ANOVA_		*	NS	**	**

† Mean values (*n* = 3) in each column followed by the same lower-case letter do not differ significantly at *p* ≤ 0.05 by Student’s *t* test—or lack of comparison of some means is because there is an interaction of the main factors. § NS, *, or **, are non-significant at *p* ≤ 0.05, significant at *p* ≤ 0.05, or *p* ≤ 0.01, respectively. C: grape marc compost, Pe: perlite, Pu: pumice, and S: soil.

**Table 3:** Concentration (mg/kg) of heavy metals and nutrient content of substrate components.

	Cu *	Pb *	Ni *	Mn *	Zn *	Fe *	N	P-Olsen	K*_exch_*	Na*_exch_*
Soil	0.159	0.459	0.038	1.222	0.19	0.74	0.091	11.52 **	60 **	380 **
Grape marc compost	0.248	0.759	0.045	0.428	0.213	4.646	2.814	0.5	1.96	0.16

Cu = copper; Pb = lead; Ni = nickel; Mn = manganese; Zn = zinc; Fe = iron; N = nitrogen; P = phosphorus; K = potassium; Na = sodium. * Heavy metal concentrations extractable by DPTA; Total concentration (%) of nutrients presented, excepting ** (mg/kg).

**Table 4:** The effect of the experimental factors, i.e., cultivation site (second floor urban roof, ground level by the side of a moderate traffic street), substrate type (3C:3Pe:4Pu, 3C:3Pe:2Pu:2S, *v*/*v*), and washing (washed, unwashed) on the concentration (mg/ kg dry matter) of the heavy metals copper (Cu), lead (Pb), nickel (Ni), manganese (Mn), zinc (Zn), and iron (Fe), in leaves and flowers of *O. dictamnus*, collected after eight-month cultivation in plastic containers with a green roof infrastructure fitted.

Three-Way ANOVA	Leaves						Flowers					
	Cu	Pb	Ni	Mn	Zn	Fe	Cu	Pb	Ni	Mn	Zn	Fe
Roof	5.7 b ^z^	17.9	4.3 b	33.3	32.4	172.0	5.1 b ^z^	18.6	5.0 a	31.6	28.4	103.9
Street	20.8 a	28.9	6.2 a	37.6	88.6	436.2	11.9 a	19.3	6.0 a	33.9	46.5	206.5
3C:3Pe:4Pu	12.7 a	22.5 a	5.0 a	29.9 b	58.7 a	261.1 b	8.5 a	18.2 a	6.3 a	27.7	36.9	166.3 a
3C:3Pe:2Pu:2S	13.8 a	24.2 a	5.5 a	41.0 a	62.2 a	347.1 a	8.6 a	19.7 a	4.7 a	37.8	37.9	144.1 a
Washed	11.9 b	22.5	4.7 b	35.0 a	54.1	225.6	8.0 a	18.1	5.4 a	32.5	32.4	124.8
Unwashed	14.5 a	24.2	5.8 a	35.9 a	66.9	382.6	9.1 a	19.8	5.6 a	33.0	42.4	185.6
Significance ^§^												
*F*_cultivation site_	**		**				**		NS			
*F*_substrate type_	NS	NS	NS	**	NS	*	NS	NS	NS			NS
*F*_washing_	*		**				NS		NS			
*F*_cult. site × substr. type_	NS	NS	NS	NS	NS	NS	NS	NS	NS	NS	*	NS
*F*_cult. site × washing_	NS	*	NS	*	**	*	NS	**	NS	*	**	*
*F*_substr. type × washing_	NS	NS	NS	NS	NS	NS	NS	NS	NS	*	NS	NS
*F*_cultivation site × substrate type × washing_	NS	NS	NS	NS	NS	NS	NS	NS	NS	**	NS	NS

^z^ Mean comparison in columns within each main factor with Student’s *t* test at *p* ≤ 0.05; means followed by the same letter are not significantly different at *p* ≤ 0.05—or lack of comparison of some means is because there is an interaction of the main factors. ^§^ NS, *, or **, are non-significant at *p* ≤ 0.05, significant at *p* ≤ 0.05, or *p* ≤ 0.01, respectively. C: grape marc compost, Pe: perlite, Pu: pumice, and S: soil.

**Table 5:** Effect of cultivation site and substrate type on concentration (mg/ kg dry matter) of the heavy metals copper (Cu), lead (Pb), nickel (Ni), manganese (Mn), zinc (Zn), and iron (Fe), in leaves (A) and flowers (B) of *O. dictamnus*, collected after cultivation for eight months in plastic containers with a green roof infrastructure fitted. Data for washed (W) and unwashed (UW) leaves and flowers are presented separately.

**A. LEAVES**							
**Cultivation Site**	**Substrate Type (*v*/*v*)**	**Cu** **W/UW**	**Pb** **W/UW**	**Ni** **W/UW**	**Mn** **W/UW**	**Zn** **W/UW**	**Fe** **W/UW**
Roof	3C:3Pe:4Pu	5.6 b ^†^/ 5.9 b	16.9 b ^z^/ 15.8 c ^z^	4.0 b ^z^/ 3.8 c ^z^	32.6 bc ^z^/ 25.3 c	35.4 b/ 33.1 b	113.3 c/ 137.1 b
	3C:3Pe:2Pu:2S	5.8 b/ 5.6 b	19.0 b ^z^/ 19.8 b ^z^	4.0 b ^z^/ 5.4 b ^z^	36.6 ab ^z^/ 38.8 ab ^z^	32.0 b/ 29.1 b	168.9 bc/ 268.8 b
Street	3C:3Pe:4Pu	15.4 a/ 23.8 a	26.5 a ^z^/ 31.0 a ^z^	5.5 a ^z^/ 6.9 a ^z^	28.5 c/ 33.4 b ^z^	69.8 a ^z^/ 96.7 a ^z^	276.3 ab/ 517.8 a
	3C:3Pe:2Pu:2S	21.0 a/ 22.9 a	27.7 a ^z^/ 30.3 a ^z^	5.4 a ^z^/ 7.1 a ^z^	45.4 a ^z^/ 46.1 a ^z^	79.2 a ^z^/ 108.7 a ^z^	343.9 a/ 606.8 a
Significance ^§^							
*F*_cultivation site_		**/ **	**/ **	**/ **	-/ **	**/ **	**/ **
*F*_substrate type_		NS/ NS	NS / NS	NS/ NS	-/ **	NS/ NS	NS/ NS
*F*_interaction_		NS/ NS	NS/ NS	NS/ NS	*/ NS	NS/ NS	NS/ NS
*F*_one-way ANOVA_		**/ **	**/ **	**/ **	**/ **	**/ **	**/ **
**B. FLOWERS**							
**Cultivation Site**	**Substrate Type (*v*/*v*)**	**Cu** **W/UW**	**Pb** **W/UW**	**Ni** **W/UW**	**Mn** **W/UW**	**Zn** **W/UW**	**Fe** **W/UW**
Roof	3C:3Pe:4Pu	5.1 b ^†^/ 4.9 b	17.6 a ^z^/ 20.0 a ^z^	6.5 a ^z^/ 4.6 a ^z^	31.7 bc ^z^/ 22.9 c	30.6 c/ 29.6 b	101.0 a/ 111.2 b
	3C:3Pe:2Pu:2S	5.2 b/ 5.1 b	20.8 a ^z^/ 19.0 a ^z^	4.1 a ^z^/ 4.9 a ^z^	34.0 ab ^z^/ 37.7 a ^z^	24.8 d/ 28.5 b	80.3 a/ 123.3 b
Street	3C:3Pe:4Pu	10.2 a/ 13.6 a	17.4 a ^z^/ 20.9 a ^z^	6.7 a ^z^/ 7.6 a ^z^	25.7 c/ 30.5 b ^z^	35.2 b/ 52.2 a	170.8 a/ 283.4 a
	3C:3Pe:2Pu:2S	11.4 a/ 12.6 a	16.5 a ^z^/ 22.5 a ^z^	4.5 a ^z^/ 5.4 a ^z^	38.5 a ^z^/ 40.8 a ^z^	39.0 a/ 59.3 a	148.2 a/ 224.6 a
Significance ^§^							
*F*_cultivation site_		**/ **	NS/ *	NS/ NS	-/ **	-/ **	*/ **
*F*_substrate type_		NS/ NS	NS/ NS	NS/ NS	-/ **	-/ NS	NS/ NS
*F*_interaction_		NS/ NS	NS/ NS	NS/ NS	*/ NS	**/ NS	NS/ NS
*F*_one-way ANOVA_		**/ **	NS/ NS	NS/ NS	**/ **	**/ **	NS/ **

^†^ Mean values (*n* = 3) in each column followed by the same lower-case letter do not differ significantly at *p* ≤ 0.05 by Student’s *t* test; these comparisons are for W and UW separately—or lack of comparison of some means is because there is an interaction of the main factors. ^z^ Mean concentration of the heavy metal higher than maximum acceptable limit for it according to FAO/ WHO [44]. ^§^ NS, *, or **, are non-significant at *p* ≤ 0.05, significant at *p* ≤ 0.05, or *p* ≤ 0.01, respectively. C: grape marc compost, Pe: perlite, Pu: pumice, and S: soil.

**Table 6:** Effect of cultivation site and substrate type on concentration (mg/ kg dry matter) of the heavy metals copper (Cu), lead (Pb), nickel (Ni), manganese (Mn), zinc (Zn), and iron (Fe), in roots of *O. dictamnus*, collected after cultivation for eight months in plastic containers with a green roof infrastructure fitted.

Cultivation Site	Substrate Type (*v*/*v*)	Cu	Pb	Ni	Mn	Zn	Fe
Roof	3C:3Pe:4Pu	13.3 a ^†^	20.8 a ^z^	10.0 a ^z^	54.0 b ^z^	43.1 a	440.7 b
	3C:3Pe:2Pu:2S	12.4 a	26.1 a ^z^	14.3 a ^z^	88.0 a ^z^	32.7 a	1.478.0 a
Street	3C:3Pe:4Pu	17.4 a	23.8 a ^z^	10.1 a ^z^	51.8 b ^z^	65.2 a ^z^	534.8 b
	3C:3Pe:2Pu:2S	16.9 a	24.9 a ^z^	16.2 a ^z^	81.0 a ^z^	48.8 a	1389.2 a
Significance ^§^							
*F*_cultivation site_		*	NS	NS	NS	NS	NS
*F*_substrate type_		NS	*	NS	**	NS	**
*F*_interaction_		NS	NS	NS	NS	NS	NS
*F*_one-way ANOVA_		NS	NS	NS	**	NS	**

^†^ Mean values (*n* = 3) in each column followed by the same lower-case letter do not differ significantly at *p* ≤ 0.05 by Student’s *t* test. ^z^ Mean concentration of the heavy metal higher than maximum acceptable limit for it according to FAO/ WHO [44]. ^§^ NS, *, or **, non-significant at *p* ≤ 0.05, significant at *p* ≤ 0.05, or *p* ≤ 0.01, respectively. C: grape marc compost, Pe: perlite, Pu: pumice and S: soil.

## Data Availability

Not applicable.
